# Indicators of quality of diabetes care in persons with type 2 diabetes with and without severe mental illness: a Danish nationwide register-based cohort study

**DOI:** 10.1016/j.lanepe.2022.100565

**Published:** 2022-12-16

**Authors:** Lenette Knudsen, Stine H. Scheuer, Lars J. Diaz, Caroline A. Jackson, Sarah H. Wild, Michael E. Benros, Dorte L. Hansen, Marit E. Jørgensen, Gregers S. Andersen

**Affiliations:** a Copenhagen University Hospital, Steno Diabetes Center Copenhagen, Herlev, Denmark; b Usher Institute, University of Edinburgh, Edinburgh, United Kingdom; c Biological and Precision Psychiatry, Copenhagen Research Centre for Mental Health, Mental Health Centre Copenhagen, Copenhagen University Hospital, Copenhagen, Denmark; d Department of Immunology and Microbiology, Faculty of Health and Medical Sciences, University of Copenhagen, Copenhagen, Denmark; e Steno Diabetes Center Greenland, Nuuk, Greenland; f National Institute of Public Health, University of Southern Denmark, Copenhagen, Denmark

**Keywords:** Quality of care, Type 2 diabetes, Comorbidity, Severe mental illness

## Abstract

**Background:**

This study aims to examine quality of diabetes care in persons with type 2 diabetes with and without severe mental illness (SMI).

**Methods:**

In a nationwide prospective register-based study, we followed persons with type 2 diabetes in Denmark with and without SMI including schizophrenia, bipolar disorder, or major depression. Quality of care was measured as receipt of care (hemoglobin A1c, low-density lipoprotein-cholesterol and urine albumin creatinine ratio assessment and eye and foot screening) and achievement of treatment targets between 2015 and 2019. Quality of care was compared in persons with and without SMI using generalized linear mixed models adjusted for key confounders.

**Findings:**

We included 216,537 persons with type 2 diabetes. At entry 16,874 (8%) had SMI. SMI was associated with lower odds of receiving care, with the most pronounced difference in urine albumin creatinine ratio assessment and eye screening (OR: 0.55, 95% CI: 0.53–0.58 and OR: 0.37 95% CI: 0.32–0.42, respectively). Among those with an assessment, we found that SMI was associated with higher achievement of recommended hemoglobin A1c levels and lower achievement of recommended low-density lipoprotein-cholesterol levels. Achievement of recommended low-density lipoprotein-cholesterol levels was similar in persons with versus without schizophrenia.

**Interpretation:**

Compared to persons without SMI, persons with SMI were less likely to receive process of care, with the most pronounced differences in urine albumin creatinine ratio assessment and eye screening.

**Funding:**

This study was funded by 10.13039/100018562Steno Diabetes Center Copenhagen through an unrestricted grant from 10.13039/501100009708Novo Nordisk Foundation.

**Research in context**

**Evidence before this study**

In Medline, we performed a title and abstract search for all previous evidence on quality of diabetes care in persons with type 2 diabetes with and without severe mental illness (published between database inception and July 30, 2022). No language restriction was applied, and we used the following search terms in various combinations; ‘severe mental illness’, ‘schizophrenia’, ‘bipolar’, ‘major depress∗’, ‘severe depress∗’, ‘psychos∗’, ‘mani∗’, ‘type 2 diabetes’, ‘diabetes mellitus’, ‘diabetes’, ‘quality of care’, ‘process of care’, ‘care’, ‘treatment’, and ‘diabetes care’. We included studies conducted in countries with universal health care coverage, including Europe, Canada, and Australia. For studies on persons with depression, we included major or severe depression. A total of ten studies were found. Previous studies from countries with universal health care coverage have found conflicting results, with two studies reporting improved quality of care in persons with severe mental illness, one reporting no difference, and three reporting lower quality of care. Four studies reported diverse findings depending on the indicators, for example one study reported no difference in assessment of hemoglobin A1c, foot and eye screening and a higher likelihood of low-density lipoprotein-cholesterol assessment in persons with compared to persons without severe mental illness. Limitations of the previous studies included limited coverage of study populations, type, and definition of severe mental illness. In summary, studies on quality of diabetes care with all types of severe mental illness collectively and individually are limited.

**Added value of this study**

This study is a nationwide study providing additional evidence on receipt of diabetes care and achievement of treatment targets in persons with type 2 diabetes with and without severe mental illness. The study addresses previous gaps by providing population-based data for persons with any severe mental illness and additionally for persons with schizophrenia, bipolar disorders, and major depression.

**Implications of all the available evidence**

Our results signify need for a change in clinical practice and health policies to reduce the gap in quality of diabetes care in persons with severe mental illness compared to persons without.

## Introduction

Compared to the background population, persons with severe mental illness (SMI), such as schizophrenia, bipolar disorder, and major depression have a 10–15-year shorter life expectancy.^1^ This may partly be due to an excess risk of type 2 diabetes and cardiovascular diseases.^1^ Persons with SMI have a 2–3 times higher risk of type 2 diabetes than the background population.^2^ Among persons with type 2 diabetes, comorbid SMI is associated with a higher risk of diabetes complications and mortality compared to persons without SMI.^3^ Disparity in quality of diabetes care may partly explain these poorer outcomes in persons with SMI.^4^

International and national diabetes care guidelines have been developed to ensure high quality of diabetes care, including annual assessments of hemoglobin A1c (HbA1c) and low-density lipoprotein (LDL)-cholesterol, and careful monitoring of achievement of treatment targets to prevent diabetes complications and mortality.^5^^,^^6^ However, patient-provider and system-level barriers can result in insufficient care among those with SMI, resulting in inequalities in quality of care.^4^

Previous studies from countries with universal health care coverage have found conflicting results,^7^, ^8^, ^9^, ^10^, ^11^, ^12^, ^13^, ^14^, ^15^, ^16^ with three studies reporting worse quality of diabetes care in persons with SMI compared to persons without,^9^^,^^11^^,^^12^ while others have found similar or better quality of care in persons with SMI.^7^^,^^8^^,^^10^^,^^13^, ^14^, ^15^, ^16^ However, most studies were conducted in persons with schizophrenia,^9^^,^^11^^,^^13^ or summarised for SMI overall,^7^^,^^8^^,^^10^^,^^12^^,^^16^ with inconsistencies in which SMI diagnoses were included. SMI comprises a heterogeneous group of diagnoses and summarizing overall SMI may underestimate differences within specific SMI diagnoses. Previous studies were also limited in methodology, such as limited data coverage resulting in selected populations^7^^,^^9^^,^^12^ or a lack of complete coverage of data on quality indicators.^9^^,^^11^ Most studies examined the quality of diabetes care on receipt of care^8^^,^^9^^,^^11^, ^12^, ^13^^,^^15^^,^^16^ and many studies only examined a few indicators.^7^^,^^8^^,^^10^, ^11^, ^12^, ^13^, ^14^ In a nationwide study, we aimed to address these gaps by examining the quality of diabetes care measured as receipt of care and achievement of treatment targets in persons with type 2 diabetes with and without SMI. We also examined whether the quality of diabetes care varied by type of SMI, including schizophrenia, bipolar disorder, and major depression.

## Methods

### Study design and study population

We identified all persons with type 2 diabetes diagnosed before 2015 who were 18 years or older at the time of type 2 diabetes diagnosis and followed them to the end of 2019. The study linked person-level data with a unique personal identification number from the Danish Civil Registration System^17^ with Danish nationwide healthcare registers.^18^ Persons with type 2 diabetes were identified in a nationwide diabetes register.^19^ The register is based on an algorithm that collects data from five health registers containing diabetes-related information.^19^ Inclusion in the diabetes register includes a diabetes diagnosis in the National Patient Register,^20^ use of diabetes podiatry in the Danish National Health Service Register,^21^ purchase of any diabetes medication in the Danish National Prescription Registry,^22^ diabetes diagnosis in the Danish Adult Diabetes Registry,^5^ or an eye screening recorded in Danish Registry of Diabetic Retinopathy.^23^

### Definition of severe mental illness

Persons with SMI were identified in the Danish Psychiatric Research Register. The register contains records of all admissions to psychiatric inpatient facilities since 1969 and visits to outpatient and emergency psychiatric departments since 1995.^24^ Persons with SMI were defined as all persons with an inpatient, outpatient or emergency contact where the diagnosis included schizophrenia or schizophrenia spectrum disorder (ICD-10: F20-F29, ICD-8: 295.x9, 296.89, 297.x9, 298.29– 298.99, 299.04, 299.05, 299.09, 301.83), bipolar disorder (ICD-10: F30-F31, ICD-8: 296.19, 296.39, 298.19) or major depression (ICD-10: F32-F33, ICD-8: 296.09, 296.29, 298.09, 300.49) from 1969 (when the register started) to 31.12.2019 (end of follow-up). There has been a lack of consensus in research of which diagnosis SMI includes. However, in most research SMI is defined as schizophrenia and schizophrenia spectrum disorder, bipolar disorder, and major depression.^25^ These diagnoses are also used in previous register-based studies from Denmark.^3^^,^^26^ The date of onset of SMI was defined as the date of first contact (inpatient, outpatient, or emergency department visit). SMI were grouped into any SMI, and each specific SMI diagnosis (schizophrenia, bipolar disorder, or major depression, which were not mutually exclusive).

### Quality of diabetes care

Quality of diabetes care was measured according to Danish National Diabetes Care Guidelines.^27^ The quality of diabetes care was measured as receipt of care in the entire population and achievement of treatment targets was measured among those who had an assessment. Receipt of care was measured as having had an assessment of HbA1c, LDL-cholesterol, urine albumin creatinine ratio (UACR), and foot- and eye screening. Achievement of recommended treatment targets among those who had an assessment was defined on the basis of HbA1c ≤53 mmol/mol, LDL-cholesterol levels ≤2.5 mmol/l, and HbA1c >70 mmol/mol. Table 1 lists the definitions of the quality of care indicators and the data sources used for each indicator. Danish national guidelines recommended that persons with diabetes should receive an assessment of HbA1c, LDL-cholesterol, UACR, and foot screening at least once every year, and eye screening once every two years in the study period.^27^ We added three months to the intervals to allow for a buffer in accordance with the national quality database.^28^ This resulted in four 15-month intervals for HbA1c, LDL-cholesterol, UACR, and foot screening and two 27-month intervals for eye screening during the five-year follow-up. We examined assessment of each indicator in each non-overlapping interval. The end of follow-up was 31.12.2019 for all indicators except for eye screening, where end of follow-up was 30.06.2019.

**Table 1:** Definition of quality indicators for diabetes care and data sources.

Quality indicators	Definition of indicators	Interval	Data sources
**Receipt of care**	**Annual assessment of HbA1c** Numerator: Persons with a HbA1c assessment Denominator: Persons with type 2 diabetes with and without SMI^a^	15 months	DADR NLD DNHSR
	**Annual assessment of LDL-cholesterol** Numerator: Persons ≥30 years with a LDL-cholesterol assessment Denominator: Persons ≥30 years old with type 2 diabetes with and without SMI^b^	15 months	DADR NLD
	**Annual assessment of UACR** Numerator: Persons with a UACR assessment Denominator: Persons with type 2 diabetes with and without SMI^a^	15 months	DADR NLD
	**Annual foot screening** Numerator: Persons with a foot screening Denominator: Persons with type 2 diabetes with and without SMI	15 months	DADR DNHSR
	**Eye screening every second year** Numerator: Persons with an eye screening Denominator: Persons with type 2 diabetes with and without SMI	27 months	DADR DNHSR Diabase
**Achievement of the treatment target**	**Recommended HbA1c levels** Numerator: Persons with HbA1c levels ≤53 mmol/mol Denominator: Persons with an assessment of HbA1c with type 2 diabetes with and without SMI^a^	15 months	DADR NLD
	**High HbA1c levels** Numerator: Persons with HbA1c levels ≥70 mmol/mol Denominator: Persons with an assessment of HbA1c with type 2 diabetes with and without SMI^a^	15 months	DADR NLD
	**Recommended LDL-cholesterol levels** Numerator: Persons ≥30 years with LDL-cholesterol levels ≤2.5 mmol/l Denominator: Persons ≥30 years with an assessment^b^	15 months	DADR NLD

HbA1c = Hemoglobin A1c; SMI = severe mental illness; LDL-cholesterol = low-density lipoprotein cholesterol; UACR = Urine albumin creatinine ratio; DADR = The Danish Adult Diabetes Registry; NLD = the National Laboratory Database; DNHSR = the Danish National Health Service Registry; Diabase = The Danish Registry of Diabetic Retinopathy.

a Population excluding the Central Denmark Region.

b Population ≥30 years excluding the Central Denmark Region.

Persons were followed from 01.01.2015 until the end of follow-up, death, or emigration, whichever came first. We excluded persons who died or emigrated within the first interval.

Data on the quality of diabetes care were obtained from the following four registers: the National Laboratory Database, which contains routine biomarker results since 2015 from all hospitals and general practitioners in all regions except the Central Denmark Region^29^; the Danish National Health Service Registry,^21^ which contains information on the use of health care services for all persons living in Denmark since 1990 and from which we used service codes related to HbA1c assessment, foot- and eye screening of persons with diabetes; the Danish Adult Diabetes Registry, containing information on the quality of diabetes care in persons with diabetes treated in outpatient clinics and general practice since 2004^5^; and the Danish Registry of Diabetic Retinopathy containing information on retinopathy screening from all hospital eye departments and private ophthalmological practices since 2013.^23^

**Fig. 1:**
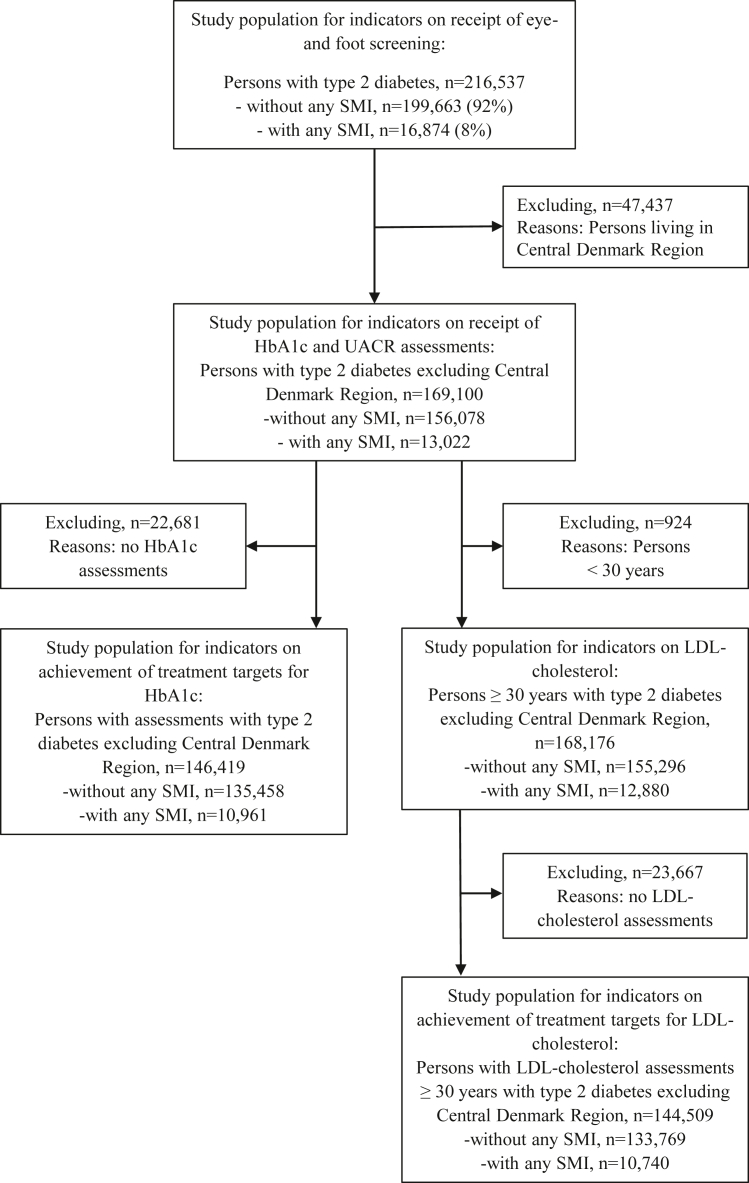
**Flowchart of study populations for each quality indicator.** SMI = severe mental illness; HbA1c = Hemoglobin A1c; LDL-cholesterol = low-density lipoprotein cholesterol; UACR = Urine albumin creatinine ratio.

As the National Laboratory Database did not include information on persons living in Central Denmark Region, we excluded that population from the analyses of quality indicators based on information from the National Laboratory Database including HbA1c, LDL-cholesterol, and UACR. A flowchart of the different study populations used for each quality indicator is presented in Fig. 1.

### Definition of covariates

We used prior evidence and the method of directed acyclic graphs to identify potential confounders and mediators (Supplementary Fig. S1). The identified potential confounders were: Age, sex, calendar time, diabetes duration (as time since date of diagnosis until time of follow-up), level of education, and migrant status. Data on date of birth, sex, and migrant status, including immigrants and refugees, was obtained from the Danish Civil Registration System.^17^ Migrants were defined as persons born outside Denmark or with parents born outside Denmark and without Danish citizenship and categorized as Danish, Western, or Non-Western.^17^ Information on the highest level of education was collected from the Danish Education Registry and defined as the highest achieved education at the date of type 2 diabetes diagnosis.^30^ It was categorized as low (lower secondary and below), medium (upper secondary), and high (tertiary and above) according to the International Standard Classification of Education.

### Statistical analysis

Characteristics of persons at the start of follow-up were presented as mean (± standard deviation [SD]) for continuous variables and as percentages (count) for categorical variables for persons with type 2 diabetes with or without any SMI, and for persons with type 2 diabetes with or without schizophrenia, bipolar disorder, or major depression, respectively.

Mixed logistic regression models were used to examine the association between the quality indicators and SMI. The value of each repeated measure of the quality indicators was included as the outcome (0/1). The models were analyzed with a person-specific random intercept to account for the correlation between the repeated measures of the quality indicators from the same person. SMI and covariates were included as fixed effects. The models were adjusted for confounders in two steps. Model 1) included basic demographic factors, age, sex, diabetes duration, and calendar time, and model 2) additionally included socio-demographic factors, education, and migrant status. SMI was included as a time-varying variable, meaning that persons were considered unexposed to SMI until a diagnosis of SMI during follow-up and then considered exposed to SMI afterwards. As the SMI groups were not mutually exclusive, we ran separate models for each SMI (any SMI, schizophrenia, bipolar disorder, and major depression). Results from models with linear versus spline terms for each continuous variable (age and diabetes duration) were compared. The results from the different models were similar, and therefore we included a linear term for each continuous variable in the final models.

The adjusted odds ratio derived from logistic regression analysis may overestimate the risk ratio when the outcome is frequent.^31^ In our study, several of the outcomes were frequent (e.g., mean HbA1c assessments was 87% in persons without SMI). To compensate for that, we also calculated the absolute risk (defined as the model-derived probability of an event) of each quality indicator for a given set of covariates.

We conducted a complete case analysis, and therefore excluded 9% of our study population due to missing information on education.

Statistical analyses were performed using R, version 4.0.2 (R Foundation for Statistical Computing, Vienna, Austria; www.R-project.org).

### Ethics

Register-based studies do not require ethical approval in Denmark. The Danish Data Protection Agency has granted access to, and use of data, and all data were anonymized.

### Data statement

All study data are held at Statistics Denmark's servers and are confidential due to privacy reasons. Access to data requires application and permission from the registries.

### Role of funding source

This study was funded by Steno Diabetes Center Copenhagen through an unrestricted grant from Novo Nordisk Foundation.

## Results

We followed 216,537 persons with type 2 diabetes; of whom 16,874 (8%) had any SMI, 12,155 (6%) major depression, 6080 (3%) schizophrenia, and 2259 (1%) bipolar disorders (flowchart presented in Fig. 1). Of those with any SMI, 15,176 (90%) were diagnosed with any SMI at start of follow-up, while 1698 (10%) were diagnosed with any SMI during follow-up and a total of 11,747 (70%) received the diagnosis before or on the same date as the type 2 diabetes diagnosis.

Of all persons with any SMI, 72% (12,155) were diagnosed with major depression, 36% (6080) with schizophrenia, and 13% (2259) with bipolar disorder.

Persons with any SMI, schizophrenia, or major depression were more likely to be younger, women, have lower education, and be of non-Western descent than persons without any SMI, schizophrenia, or major depression, respectively (Table 2). Persons with bipolar disorder were also more likely to be younger, women, but had similar education levels and migration status, compared to persons without (Table 2).

**Table 2:** Characteristics of persons with any SMI, schizophrenia, bipolar disorder, major depression or without any SMI, schizophrenia, bipolar, and major depression at the start of follow-up.

	Without any SMI (n = 199,663)	Any SMI (n = 16,874)	Schizophrenia (n = 6080)	Bipolar disorder (n = 2259)	Major depression (n = 12,155)
Age at start of follow-up, mean (±SD) years	66.7 (12.2)	62.2 (13.5)	59.6 (13.2)	63.0 (12.0)	63.0 (13.6)
Women, no. %	88,863 (44.5)	9347 (55.4)	3171 (52.2)	1288 (57.0)	7069 (58.2)
Diabetes duration at start of follow-up, median (IQR)	6.2 [3.2; 11.4]	5.9 [3.0; 11.1]	6.0 [3.1; 11.2]	6.0 [3.2; 11.0]	5.9 [3.0; 11.1]
Education at type 2 diabetes diagnosis, no. (%)					
Low	76,066 (38.1)	7414 (48.6)	2983 (49.1)	870 (38.6)	5149 (42.4)
Medium	75,454 (37.8)	5413 (35.5)	1744 (28.7)	789 (34.9)	4010 (33.0)
High	29,262 (14.7)	2441 (16.0)	742 (12.2)	434 (19.2)	1869 (15.4)
Missing, no (%)	18,881 (9.5)	1606 (9.5)	611 (10.0)	166 (7.3)	1127 (9.2)
Migrant status, no. (%)					
Danish	177,237 (88.8)	14,463 (85.7)	5114 (84.1)	2074 (91.8)	10,483 (86.3)
Western decent	4901 (2.5)	436 (2.6)	151 (2.5)	66 (2.9)	307 (2.5)
Non-Western decent	17,525 (8.8)	1975 (11.7)	815 (13.4)	119 (5.3)	1365 (11.2)
Type of SMI, no. (%)					
Schizophrenia		6080 (36.0)	6080 (100.0)	883 (39.1)	1952 (16.1)
Bipolar disorder		2259 (13.4)	883 (14.5)	2259 (100.0)	1251 (10.3)
Major depression		12,155 (72.0)	1952 (32.1)	1251 (55.4)	12,155 (100.0)
Receipt of care during the entire follow-up:					
HbA1c assessment, mean (±SD)^a^	0.87 (0.34)	0.85 (0.36)	0.84 (0.36)	0.85 (0.36)	0.85 (0.35)
UACR assessment, mean (±SD)^a^	0.55 (0.50)	0.46 (0.50)	0.42 (0.49)	0.43 (0.49)	0.48 (0.50)
LDL-cholesterol assessment, mean (±SD)^b^	0.81 (0.39)	0.78 (0.41)	0.78 (0.42)	0.79 (0.41)	0.79 (0.41)
Foot screening, mean (±SD)	0.50 (0.50)	0.46 (0.50)	0.46 (0.50)	0.50 (0.50)	0.46 (0.50)
Eye screening, mean (±SD)	0.67 (0.47)	0.56 (0.50)	0.53 (0.50)	0.54 (0.50)	0.57 (0.49)
Achieving treatment targets in persons with assessments during the entire follow-up					
HbA1c ≤53 mmol/mol, mean (±SD)^c^	0.59 (0.49)	0.60 (0.49)	0.61 (0.49)	0.65 (0.48)	0.60 (0.49)
HbA1c ≥70 mmol/mol, mean (±SD)^c^	0.13 (0.33)	0.15 (0.36)	0.16 (0.36)	0.12 (0.33)	0.15 (0.36)
LDL-cholesterol ≤2.5 mmol/l, mean (±SD)^d^	0.76 (0.43)	0.72 (0.45)	0.73 (0.44)	0.72 (0.45)	0.71 (0.46)

SMI = Severe mental illness; HbA1c = Hemoglobin A1c; LDL-cholesterol = low-density lipoprotein cholesterol; UACR = Urine albumin creatinine ratio; SD = Standard deviation; IQR = Interquartile range.

a Population excluding the Central Denmark Region (n = 169,100).

b Population ≥30 years excluding the Central Denmark Region (n = 168,176).

c Among the population with assessments excluding the Central Denmark Region: without any SMI n = 135,458 (87% of the population), with any SMI n = 10,961 (84% of the population), with schizophrenia n = 4066 (83% of the population), with bipolar disorder n = 1396 (82% of the population), with major depression n = 7837 (85% of the population).

d Among the population with assessments ≥30 years excluding the Central Denmark Region: without any SMI n = 133,769 (86% of the population), with any SMI n = 10,740 (83% of the population), with schizophrenia n = 3954 (82% of the population), with bipolar disorder n = 1376 (81% of the population), with major depression n = 7693 (84% of the population).

**Fig. 2:**
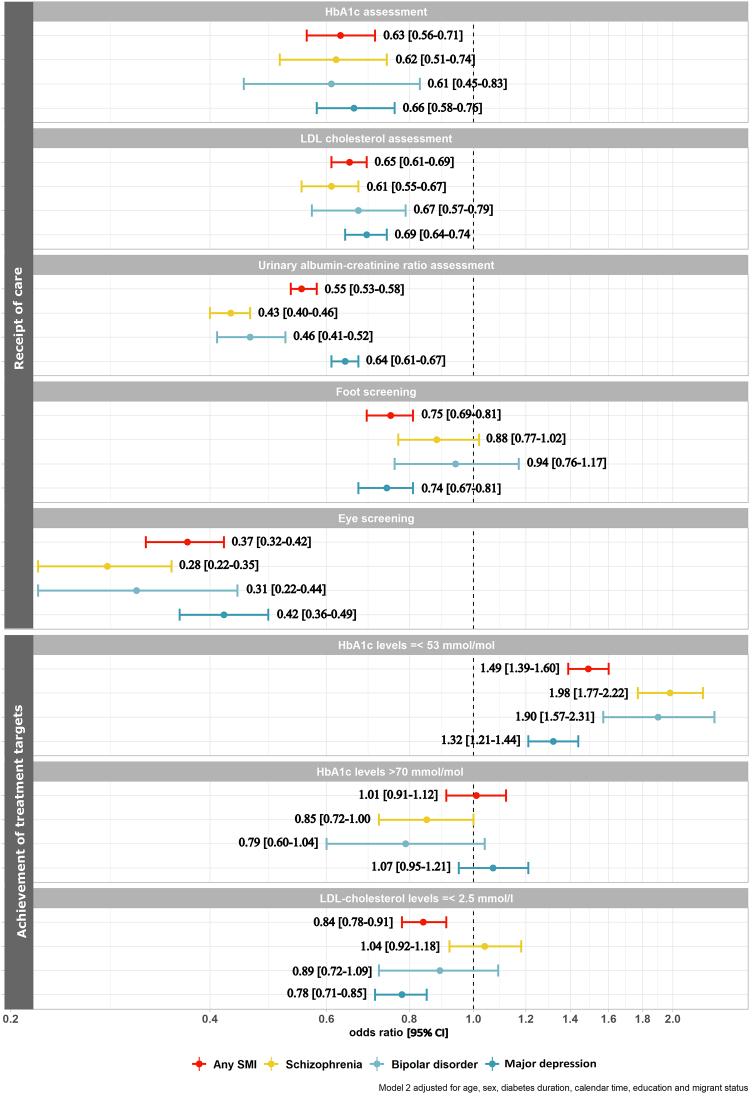
**Odds Ratios (95% CI) for receipt of care and achievement of treatment targets in persons with any SMI, schizophrenia, bipolar disorder, or major depression compared to persons without any SMI, schizophrenia, bipolar disorder, or major depression, respectively (model 2∗).** ∗Model 2 adjusted for age, sex, diabetes duration, calendar time, education, and migrant status. † In persons with assessments. SMI = Severe mental illness; HbA1c = Hemoglobin A1c; LDL-cholesterol = low-density lipoprotein cholesterol; UACR = Urine albumin creatinine ratio; CI = confidence interval.

Differences in receipt of care and achievement of treatment targets over the entire follow-up adjusted for confounders are presented in Fig. 2.

### Receipt of care

Persons with any SMI, schizophrenia, bipolar disorder, and major depression had lower odds of receiving HbA1c, LDL-cholesterol, UACR assessments, and eye screenings than persons without the specific SMI (Fig. 2). We found the lowest odds for UACR assessments and eye screenings (results for any SMI: OR: 0.55, 95% CI: 0.53–0.58 and OR: 0.37, 95% CI: 0.32–0.42, respectively).

The odds of receipt of assessments of HbA1c and LDL-cholesterol were similar across the different SMI diagnoses, whereas it differed for UACR and eye screening. For UACR assessments and eye screenings, the effect was greater for persons with schizophrenia and bipolar disorder compared to persons with major depression. Persons with any SMI or major depression had lower odds of receiving foot screening than those without. This was also the case with schizophrenia or bipolar disorder, albeit the latter analyses did not reach statistical significance.

The absolute risk for persons with fixed covariates was 45.1% vs. 59.7% for UACR assessment and 69.5% vs. 75.3% for foot screening in persons with vs. without any SMI. The absolute risk for LDL-cholesterol was 92.6% vs. 95.1% in persons with vs. without any SMI, whereas it was close to one for both HbA1c assessment and eye screening (e.g., the absolute risk for eye screening was 99.8% in persons with any SMI and 99.9% in persons without SMI) (absolute risks are presented in Supplementary Table S3).

### Achievement of treatment targets

Among persons who had an assessment, any SMI, schizophrenia, bipolar disorder, or major depression were associated with higher odds of achieving HbA1c targets. Compared to persons without, persons with schizophrenia or bipolar disorders had the highest odds of having HbA1c ≤53 mmol/mol (OR 1.98, 95% CI: 1.77–2.22; OR 1.90, 95% CI: 1.57–2.31, respectively). We found no differences in odds of HbA1c >70 mmol/mol in persons with any SMI or major depression compared to persons without the specific SMI. In contrast, we found lower odds of HbA1c >70 mmol/mol in persons with schizophrenia or bipolar disorders than in those without, however, the confidence intervals included 1 (OR 0.85 [0.72–1.00]; OR 0.79 [0.60–1.04] respectively).

In persons who had an assessment, persons with any SMI or major depression alone had lower odds of LDL-cholesterol ≤2.5 mmol/l (OR 0.84, 95% CI: 0.78–0.91; OR 0.78, 95% CI: 0.71–0.85, respectively) compared to persons without, while we found no difference for persons with bipolar disorder or schizophrenia when compared to persons without the specific SMI.

Adjustment for potential confounders only slightly changed the effect estimates (results of model 1 are shown in Supplementary Table S2, and results of model 2 are shown in Fig. 2).

The absolute risk for the treatment target HbA1c ≤53 mmol/mol was 79.4% vs. 72.1% in persons with vs. without any SMI and for HbA1c >70 mmol/mol it was 0.5% in both persons with and without any SMI. For LDL-cholesterol the absolute risk was 89.9% vs. 91.4% in persons with vs. without any SMI (absolute risks are presented in Supplementary Table S3).

## Discussion

### Main findings

In this nationwide prospective follow-up study, we found that persons with SMI had markedly lower receipt of HbA1c, LDL-cholesterol, UACR assessments, and eye screenings compared to persons without SMI. The difference was most pronounced for UACR assessment and eye screening, where persons with SMI had 45% and 63% lower odds of receiving assessments of UACR or eye screening, respectively.

Among persons with an assessment, we found that persons with SMI had higher achievement of recommended HbA1c levels, while they had a lower achievement of recommended LDL-cholesterol levels compared to persons without SMI. However, some of the results differed when comparing persons with and without schizophrenia or bipolar disorders. For example, persons with schizophrenia had no difference in achieving recommended LDL-cholesterol levels compared to persons without schizophrenia.

For HbA1c assessment and eye screening and to some extent also LDL-cholesterol assessment there was a very high coverage of assessments and screenings both in persons with and without SMI (absolute risks were close to one), suggesting that the lower odds from the logistic regression may exaggerate a risk association.^31^ Thus, the results related to these indicators may be of limited clinical importance.

The revealed inequalities in receiving care in persons with SMI could be due to patient-provider level barriers. In periods with severe psychiatric symptoms, physical health often comes second, both among professionals and persons with diabetes.^4^

The treatment of SMI and diabetes in two compartmentalised health systems might contribute to more barriers in offering a routine follow-up to persons with diabetes. In Denmark, 80% of persons with type 2 diabetes have a general practitioner as their primary diabetes health professional, and the remaining persons with more complex treatment courses receive care in endocrinological outpatient clinics.^32^

The diabetes health professionals are responsible for ensuring annual assessment of HbA1c, LDL-cholesterol, UACR, and foot- and eye screening among persons with diabetes. The diabetes health professionals prescribes an annual assessment of HbA1c, LDL-cholesterol, and UACR at a laboratory. The diabetes health professionals do encourage their patients to book an appointment for foot- and eye screening, but the person with diabetes have to book appointments with the podiatrist and ophthalmologist themselves. The cost of foot screenings is partly subsidized, and ophthalmologists often have long waiting times. Mental health services in Denmark are responsible for annual assessment of HbA1c and LDL-cholesterol among persons receiving active psychiatric treatment who have not already received this in primary care. This is to monitor for side effects of the psychiatric treatment.

More pronounced difference for UACR and eye screening among persons with SMI could therefore be due to the additional barriers in obtaining these assessments. UACR assessment obviously requires the individual to collect a urine sample, which persons often find unpleasant or difficult and needs extra encouragement from the health professionals. Persons with SMI may face more challenges with providing the urine sample or the diabetes health professional may be more reluctant to encourage sample collection in this group. Eye screening is conducted by an ophthalmologist, which could be far away from the persons’ home and the persons will have to book the appointment themselves. Persons with SMI may be less willing to receive care in a less familiar setting and to book and remember to attend the appointment themselves.

We found that among persons with assessments, those with SMI were more likely to have recommended HbA1c levels. These findings could be because a lower proportion with SMI received care in the first place. It is likely, that a smaller proportion receiving care often results in improved achievement of treatment targets, as the persons receiving care may be healthier than persons not receiving care. Another possible explanation could be that both diabetes and psychiatric health professional pay attention to and react to the results of the HbA1c assessments. On the other hand, we found that any SMI and major depression were associated with lower achievement of recommended LDL-cholesterol.

We found a difference in receipt of diabetes care and achievement of treatment targets across SMI diagnoses highlighting the importance of analyzing each diagnosis separately. The difference may indicate diverse awareness or barriers within different diagnoses. However, the reasons need to be explored further and addressed.

### Comparison with previous studies

In this study of persons with type 2 diabetes, we found that 8% had co-existing SMI, 6% major depression, 3% schizophrenia, and 1% bipolar disorder. The prevalence was higher in our study compared to a Scottish study reporting that of all persons with type 2 diabetes, 1%, 0.5%, and 3% had a hospital admission for respectively schizophrenia, bipolar disorder, or major depression.^15^ The higher prevalence in our study is likely due to the inclusion of both in and out-patient contacts. Opposite this, a systematic review found that the prevalence of depression was 18% in persons with type 2 diabetes^33^ However, they included mild, moderate, and major depression, whereas we only included major depression, which can explain the differences in prevalence.

### Receipt of care

In line with our results, previous studies have reported a lower receipt of care for assessments of HbA1c, LDL-cholesterol, UACR, and eye screening^11^^,^^12^ and one study found no difference in foot screening for persons with and without schizophrenia.^9^ Contrary to our findings, other studies found no difference in receipt of assessment of HbA1c,^7^^,^^9^^,^^16^ LDL-cholesterol^8^^,^^9^ and no difference^8^^,^^16^ or marginally lower odds of foot- or eye screening and receipt of UACR assessment^9^ in persons with SMI. However, one study found a higher number of LDL-cholesterol assessments in persons with SMI^16^ and another study found higher odds of UACR assessment.^16^ A recent Scottish study found that persons with SMI were more likely to receive HbA1c, LDL-cholesterol, UACR, and foot- and eye screening the first year after type 2 diabetes diagnosis compared to persons without,^15^ which is contrary to our results. However, when examining the quality of care over 10 years, persons with SMI were less likely to receive eye screening, which was in line with our results.

The difference between our results and previous studies could be due to differences in methodology, such as data sources and the definition of study populations. The definition of the SMI population differed in our study and previous studies.^7^^,^^8^^,^^16^ For example, one study defined SMI as schizophrenia or bipolar disorder whereas we also included major depression.^16^ A Scottish study only based the definition of SMI on inpatient contacts,^15^ whereas we also included outpatient contacts.

The definition of the diabetes population also differed in our study compared to previous studies. Our study included complete data for all persons with type 2 diabetes from outpatient clinics and primary care. Whereas a Scottish study only included persons with newly diagnosed type 2 diabetes,^15^ a UK study only included persons with type 2 diabetes treated in selected general practices,^16^ and a Danish study included persons with type 1 or type 2 diabetes.^9^

The differences between the Danish and Scottish studies could also be an expression of better quality of diabetes care in persons with SMI in Scotland. In Scotland, the pay-performance scheme for general practitioners offered financial incentives to promote good practice, including assessing cardiometabolic risk factors in persons with SMI.^34^ In Scotland, foot screening is expected to be performed as part of the annual review of persons with diabetes and invitations to eye screening on a specified date and in a specified place are sent to persons with diabetes, with the opportunity to change the appointment by telephone. In Denmark, general practitioners do not have the same financial incentives to promote care and persons with diabetes are expected to arrange their own foot and eye screening. However, whether the differences are due to differences in methodology or health care should be addressed in future studies.

### Achievement of treatment targets

Two previous studies found that SMI was associated with higher proportions of persons achieving good glycemic control,^7^^,^^16^ which was in line with our findings. Opposite this, one study found lower proportions achieving good glycemic control^10^ and two studies found no difference.^13^^,^^14^ In line with our findings, one previous study found that depression was associated with better achievement of lipid targets,^14^ while two other studies found no difference between persons with and without SMI.^10^^,^^16^ Two of the previous studies were based on crude data,^7^^,^^13^ whereas we controlled for possible confounders and examined repeated measures over time in mixed-effect models which could explain the differences in findings.

### Strengths and limitations

Our study has several strengths. The use of different nationwide registers made it possible to construct a nationwide prospective study with data on almost all persons in Denmark with type 2 diabetes with and without SMI, with no selection due to health coverage or participation in a survey. This means that the findings are generalizable to Denmark's entire type 2 diabetes population. The cohort of persons with type 2 diabetes is based on the diabetes register, which is constructed using five national registers.^19^ In Denmark, around 80% of persons with type 2 diabetes are treated in general practice and therefore do not have a diagnosis in the National Patient Register.^19^ However, these persons are captured in the diabetes register, as it uses diabetes-defining information from other registers such as use of podiatry in the Danish National Health Service Registry, diabetes medication in the Danish National Prescription Registry, and eye examination in the Danish Registry of Diabetic Retinopathy.^23^ Despite the strength of including persons with type 2 diabetes treated in general practice, we were not able to capture persons with undiagnosed diabetes. In Denmark, no systematic screening for type 2 diabetes exists nor for persons with SMI. Whether or not more person with SMI have undiagnosed diabetes is difficult to predict.

We used complete data on quality indicators from several registers with high coverage and high data validity.^17^^,^^19^^,^^21^^,^^24^^,^^29^^,^^30^ For example, this included data on HbA1c, LDL-cholesterol, and UACR from the National Laboratory Database, which provides information on all laboratory tests in the entire study population except for persons living in Denmark Central Region, who was excluded for these analyses. The longitudinal nature of the data allowed us to examine the quality of diabetes care over five years and account for changes over time. Moreover, we examined receipt of care and achievement of treatment targets which provided a more nuanced exploration of the quality of diabetes care, whereas previous studies have primarily focused on receipt of care.^8^^,^^9^^,^^11^^,^^12^^,^^15^^,^^16^ Additionally, we examined the quality of diabetes care in persons with type 2 diabetes with and without any SMI and specific diagnoses of SMI, which allowed us to examine differences overall and across different SMI diagnoses. Lastly, we could distinguish between the type of diabetes, thus including persons with type 2 diabetes only. Several former studies have not distinguished between type 1 and type 2 diabetes.^9^^,^^12^^,^^16^

Our study also has some limitations. Since SMI was ascertained using in- and outpatient psychiatric hospital records, we did not include persons with SMI who received a diagnosis in primary care or at a private psychologist. However, as most persons with a suspected SMI would be referred to a psychiatric hospital, we do not believe this would exclude a large proportion with SMI. Although we included persons from in- and outpatient psychiatric records, it was impossible to distinguish the ascertainment route, so we could not examine differences in quality indicators in different severity of SMI. Our study only included persons with more severe cases of depression, referred to as major depression, requiring treatment in the secondary health care sector, so the findings might not be generalizable to persons with less severe depression treated in primary care by a general practitioner or a psychologist. Potential confounders and mediators were identified using directed acyclic graphs and based on prior evidence. However, we cannot reject that a different directed acyclic graph would have changed the structure of the analyses. We excluded around 9% of our study population due to missing information on the level of education. When comparing persons with and without missing information on education, we found that persons with missing information were older, had longer duration of diabetes and were more often migrants (Supplementary Table S1). These persons might also receive a lower quality of diabetes care^30^ and thus this exclusion might have introduced selection bias, which could result in some underestimation of our findings.

There was a large proportion of missingness in achievement of treatment targets, with 13–19% of persons without any measurements during follow-up. We were only able to examine differences in persons with values of HbA1c and LDL-cholesterol, where a higher proportion with SMI had missing values. This means that we may have introduced selection bias in the results on achieving treatment targets.

Investigation of the role of the well-recognized metabolic effects of treatments for SMI was beyond the scope of this study and requires further research, particularly among persons with diabetes. Data on other important receipt of care and treatment targets including blood pressure and body mass index were not available in this study. Further research is required to address whether more stringent treatment targets for sub-groups of the study population for example persons with a history of cardiovascular disease or albuminuria were met and whether recommended lipid-lowering or diabetes treatments were prescribed appropriately.

### Conclusions

Persons with SMI had a markedly lower receipt of assessment of HbA1c, LDL-cholesterol, UACR, and eye screening, compared to persons without SMI, with the most pronounced differences for UACR and eye screening. Due to high coverage of HbA1c and LDL-cholesterol assessments and eye screening, the finding related to UACR assessments may be of highest clinical importance.

Among persons with assessments, we found that persons with SMI had better achievement of recommended levels of HbA1c and lower achievement of recommended LDL-cholesterol levels. These results may reflect persons with SMI who are healthier and have fewer complications than those who did not receive assessments.

Our findings highlight the need to develop effective interventions to reduce marked inequalities in diabetes care between persons with and without SMI. The pronounced differences could contribute to higher risk of complications and mortality in persons with diabetes and SMI compared to persons with diabetes only.

## Contributors

LK, SHS, MEB, DLH, MEJ and GSA led the conception, design, and planning of the study. LK and SHS lead data management and analyses with support from LJD and GSA. LK led drafting of the work with support from SHS. All authors contributed to the interpretation of the data and revising the manuscript critically for important intellectual content and read and approved the final manuscript. LK and SHS are responsible for the overall content of the manuscript as guarantors. LK, SHS, LJD and GSA had access to the data and LK, SHS and GSA controlled the decision to publish.

## Data sharing statement

The data used in this study are held at Statistics Denmark's servers. The data are confidential for data privacy reasons and therefore, cannot be made publicly available. Access to data requires an application and permission from the different owners of the registers.

## Prior presentation

Parts of this study were presented at the European Diabetes Epidemiology Group Annual meeting in Greece, 2nd – 5th April 2022.

## Declaration of interests

LK: holds shares in Novo Nordisk A/S, SHS: none, LJD: none, CAJ: none, SHW: none, MEB: none, DLH: None, MEJ: holds shares in Novo Nordisk, has received research grants from AMGEN, Astra Zeneca, 10.13039/100001003Boehringer Ingelheim, Novo Nordisk and Sanofi Aventis, GSA: holds shares in Novo Nordisk A/S.

## Appendix A Supplementary data

Supplementary Materials

STROBE-checklist-v4-cohort
