# Supplementary material for: Indicators of quality of diabetes care in persons with type 2 diabetes with and without severe mental illness: a Danish nationwide register-based cohort study
Source: Lancet Reg Health Eur. 2022 Dec 16;26:100565. doi: 10.1016/j.lanepe.2022.100565 (PMC9989638; doi:10.1016/j.lanepe.2022.100565)
Supplement: Supplementary Materials [file mmc1.docx]

**Supplementary Materials**


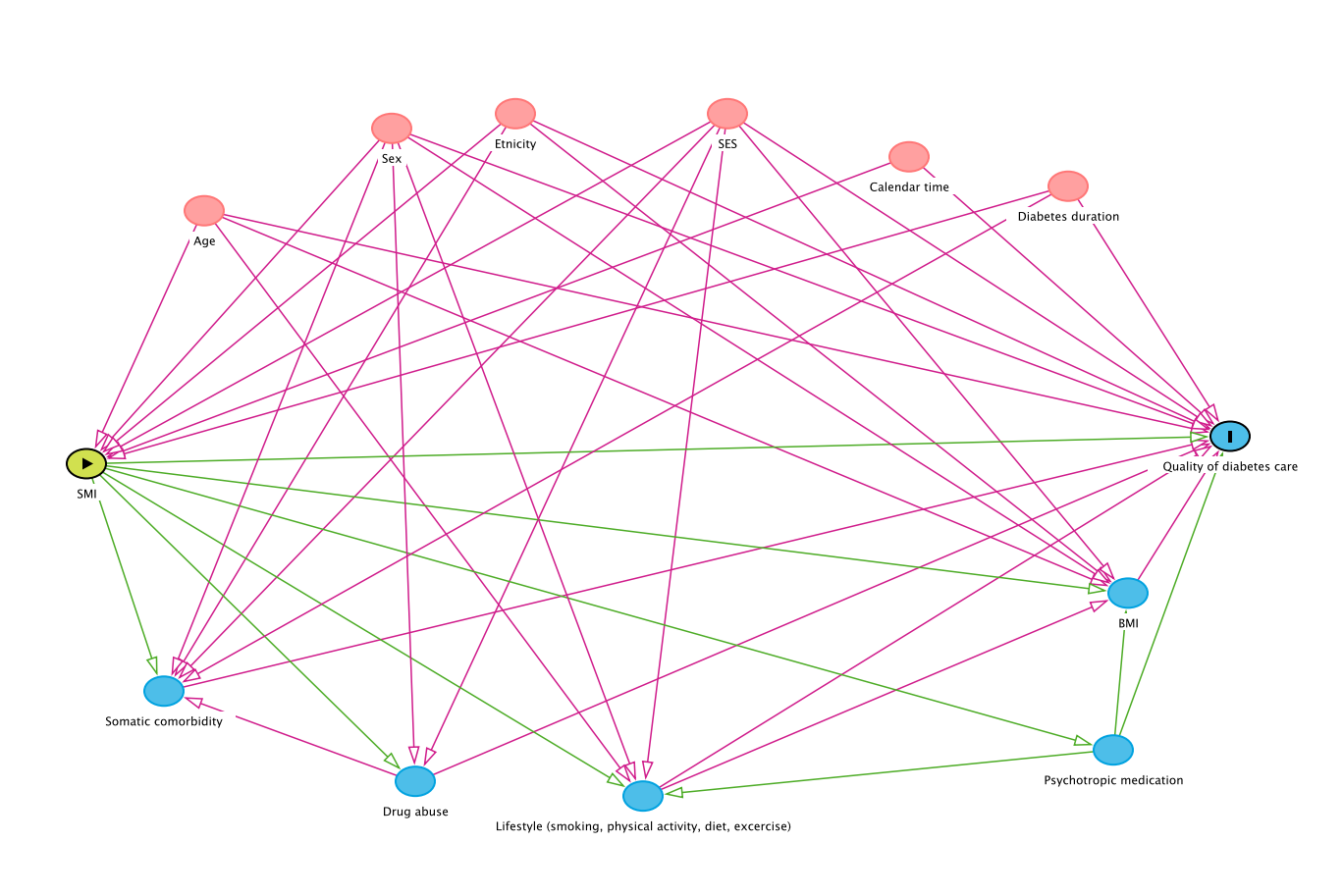


**Supplementary Figure 1**. Directed acyclic graph illustrating the causal network of association between SMI and quality of care applied to identify potential confounders. The identified confounders were age, sex, diabetes duration, calendar time, SES*, Ethnicity*, and the identified mediating factors were; lifestyle, substance use disorder, BMI, psychotropic medication, and somatic comorbidity. The analyses were not adjusted for potential mediating factors.
*Educational level used as proxy for socio-economic status and migrant status used as proxy for ethnicity

Abbreviations: SMI = Severe mental illness, BMI = body mass index, SES = socio-economic status

**Supplementary Table 1.** Difference in characteristics between persons with and without missing data on level of education

|  | Total study population (n=216,537) | Persons without missing data (n=196,050) | Persons with missing data (n=20,487) |
| --- | --- | --- | --- |
| Age at start of follow-up, mean years ((±SD) Women, no. % Diabetes duration at the start of follow-up, median (IQR) Migrant status, no. (%)  Danish  Western decent   Non-Western decent Type of SMI, no. (%)  Any SMI  Schizophrenia  Bipolar disorder  Major depression Receipt of care during the entire follow-up:  HbA1c assessment, mean (±SD)*  UACR assessment, mean (±SD)*  LDL-cholesterol assessment, mean (±SD) prevalence†  Foot screening, mean (±SD)   Eye screening, mean (±SD)  Achieving treatment targets in persons with assessments during the entire follow-up:  HbA1c ≤53 mmol/mol, mean (±SD)*  HbA1c ≥70 mmol/mol, mean (±SD)*  LDL-cholesterol ≤2.5 mmol/l, mean (±SD)† | 66.3 (12.4) 98,210 (45.4) 6.2 [3.1; 11.4]  191,700 (88.5) 5,337 (2.5) 19,500 (9.0)  16,874 (7.8) 6,080 (36.0) 2,259 (13.4) 12,155 (72.0) | 66.1 (12.2) 87,456 (44.6) 5.7 [3.0; 10.4]  176,789 (90.2) 4,569 (2.3) 14,692 (7.5)  15,268 (7.8) 5,469 (35.8) 2,093 (13.7) 11,028 (72.2)  0.87 (0.34) 0.55 (0.50) 0.81 (0.39) 0.49 (0.50) 0.66 (0.47)  0.60 (0.49) 0.12 (0.32) 0.75 (0.43) | 69.1 (13.4) 10,754 (52.5) 19.6 [9.9; 20.3]  14,911 (72.8) 768 (3.7) 4,808 (23.5)  1,606 (7.8) 611 (38.0) 166 (10.3) 1,127 (70.2)  0.83 (0.37) 0.55 (0.50) 0.77 (0.42) 0.52 (0.50) 0.64 (0.48)  0.41 (0.49) 0.22 (0.41) 0.77 (0.42) |
| *Population excluding the Central Denmark Region (n=169,100) †Population ≥ 30 years excluding the Central Denmark Region (n=168,176)  Abbreviations: SMI = Severe mental illness; HbA1c = Hemoglobin A1c; LDL-cholesterol = low-density lipoprotein cholesterol; UACR = Urine albumin creatinine ratio; SD = Standard deviation; IQR = Interquartile range | | | |

**Supplementary Table 2.** Odds Ratio (95% CI) for receipt of care and achievement of treatment targets in persons with any SMI, schizophrenia, bipolar disorder, or major depression compared to persons without any SMI, schizophrenia, bipolar disorder, or major depression, respectively†

|  | Model 0  OR (95% CI) | Model 1 OR (95% CI) |
| --- | --- | --- |
| Receipt of care during the entire follow-up:  HbA1c*  Any SMI  Schizophrenia  Bipolar disorder  Depression  LDL-cholesterol*  Any SMI  Schizophrenia  Bipolar disorder  Depression  Urinary albumin-creatinine ratio*  Any SMI  Schizophrenia  Bipolar disorder  Depression  Foot screening  Any SMI  Schizophrenia  Bipolar disorder  Depression  Eye screening  Any SMI  Schizophrenia  Bipolar disorder  Depression  Achieving treatment targets in persons with assessments  HbA1c ≤53 mmol/mol*  Any SMI  Schizophrenia  Bipolar disorder  Depression  HbA1c >70 mmol/mol*  Any SMI  Schizophrenia  Bipolar disorder  Depression  LDL-cholesterol > 2.5 mmol/l*  Any SMI  Schizophrenia  Bipolar disorder  Depression | 0.78 [0.70; 0.87]  0.77 [0.65; 0.92]  0.71 [0.53; 0.94]  0.80 [0.70; 0.91]  0.73 [0.69 ;0.77]  0.69 [0.62; 0.75]  0.71 [0.60; 0.83]  0.77 [0.72; 0.83]  0.58 [0.55; 0.60] 0.46 [0.43; 0.49] 0.48 [0.43; 0.53]  0.65 [0.62; 0.68]  0.70 [0.65; 0.74] 0.66 [0.59; 0.74] 1.00 [0.84; 1.19]  0.73 [0.67; 0.78]  0.38 [0.33; 0.42]  0.28 [0.23; 0.35]  0.34 [0.25; 0.47]  0.42 [0.37; 0.48]   1.09 [1.02; 1.17] 1.14 [1.02; 1.27] 1.71 [1.42; 2.05] 1.09 [1.01; 1.18]  1.37 [1.24; 1.51]  1.45 [1.23; 1.71]  0.96 [0.73; 1.26]  1.32 [1.18; 1.48]  0.67 [0.62; 0.71] 0.80 [0.71; 0.89] 0.77 [0.64; 0.93] 0.62 [0.57; 0.67] | 0.64 [0.57; 0.71]  0.61 [0.51; 0.73]  0.60 [0.45; 0.80]  0.67 [0.59; 0.76]  0.65 [0.61; 0.69]  0.59 [0.54; 0.65]  0.66 [0.57; 0.77]  0.70 [0.65; 0.75]  0.56 [0.53; 0.58]  0.43 [0.40; 0.46]  0.47 [0.42; 0.53]  0.64 [0.61; 0.67]  0.77 [0.71; 0.83]  0.89 [0.78; 1.01]  1.10 [0.89; 1.35]  0.75 [0.68; 0.82]  0.38 [0.34; 0.43]  0.28 [0.23; 0.35]  0.34 [0.25; 0.48]  0.43 [0.38; 0.50]   1.34 [1.25; 1.43]  1.71 [1.54; 1.90]  1.91 [1.61; 2.28]  1.21 [1.13; 1.31]  1.19 [1.07; 1.32]  0.98 [0.85; 1.13]  0.79 [0.62; 1.01]  1.19 [1.07; 1.32]  0.83 [0.78; 0.90]  1.04 [0.93; 1.16]  0.93 [0.77; 1.12]  0.77 [0.71; 0.84] |
| *Population excluding the Central Denmark Region (n=142,302)  †Model 0 – unadjusted, model 1 – adjusted for age, sex, diabetes duration and calendar time | | |

**Supplementary Table 3.** Absolute risk (defined as the model-derived probability of an event) for receipt of care and achievement of treatment targets in persons given a particular set of covariates^1^

|  | SMI | | Schizophrenia | | Bipolar disorder | | Major depression | |
| --- | --- | --- | --- | --- | --- | --- | --- | --- |
|  | No SMI | Any SMI | No schizophrenia | Schizophrenia | No bipolar disorder | Bipolar disorder | No major depression | Major depression |
| Receipt of care:  Annual assessment of   HbA1c*  LDL-cholesterol*  Urinary albumin-creatinine ratio*  Foot screening  Eye screening  Achieving treatment targets in persons with assessments  HbA1c ≤53 mmol/mol*  HbA1c >70 mmol/mol*  LDL-cholesterol > 2.5 mmol/l* | 99.98 95.06 59.73 75.25 99.92   72.12  0.53  91.36 | 99.97 92.63 45.12 69.49 99.80   79.40 0.54 89.93 | 99.98 94.95 59.10 74.77 99.92   72.35 0.54 91.93 | 99.97 91.99 38.33 72.38 99.72  83.83 0.46 91.54 | 99.98 94.88 58.53 74.69 99.92  72.70 0.54 91.25 | 99.97 92.56 39.58 73.52 99.75  83.52 0.42 90.27 | 99.98 94.99 59.07 75.12 99.92  72.49 0.53 91.37 | 99.98 92.92 48.02 68.96 99.82  77.69 0.57 89.15 |
| *Population excluding the Central Denmark Region (n=142,302)  ^1^age = 66 years, sex = women, diabetes duration = 7.8 years, calendar time = interval 4, education = lower secondary and below, migrant status = Danish  ^2^The high absolute risk for annual assessment of HbA1c and eye screening reflects that a high proportion have had annual assessment and screening every second year | | | | | | | | |
